# Increased leaf mesophyll porosity following transient retinoblastoma-related protein silencing is revealed by microcomputed tomography imaging and leads to a system-level physiological response to the altered cell division pattern

**DOI:** 10.1111/tpj.12342

**Published:** 2013-11-11

**Authors:** Carmen Dorca-Fornell, Radoslaw Pajor, Christoph Lehmeier, Marísa Pérez-Bueno, Marion Bauch, Jen Sloan, Colin Osborne, Stephen Rolfe, Craig Sturrock, Sacha Mooney, Andrew Fleming

**Affiliations:** 1 Department of Animal and Plant Sciences, University of SheffieldWestern Bank, Sheffield, S10 2TN, UK; 2 Division of Agriculture and Environmental Sciences, School of Biosciences, University of NottinghamSutton Bonington Campus, Loughborough, LE12 5RD, UK

**Keywords:** leaf, development, cell cycle, physiology, imaging, *Arabidopsis thaliana*

## Abstract

The causal relationship between cell division and growth in plants is complex. Although altered expression of cell-cycle genes frequently leads to altered organ growth, there are many examples where manipulation of the division machinery leads to a limited outcome at the level of organ form, despite changes in constituent cell size. One possibility, which has been under-explored, is that altered division patterns resulting from manipulation of cell-cycle gene expression alter the physiology of the organ, and that this has an effect on growth. We performed a series of experiments on retinoblastoma-related protein (RBR), a well characterized regulator of the cell cycle, to investigate the outcome of altered cell division on leaf physiology. Our approach involved combination of high-resolution microCT imaging and physiological analysis with a transient gene induction system, providing a powerful approach for the study of developmental physiology. Our investigation identifies a new role for RBR in mesophyll differentiation that affects tissue porosity and the distribution of air space within the leaf. The data demonstrate the importance of RBR in early leaf development and the extent to which physiology adapts to modified cellular architecture resulting from altered cell-cycle gene expression.

## Introduction

Research over the last two decades has provided fundamental insight into the plant cell cycle, identifying not only the lead genes involved in progression through the cycle but also providing a significant understanding of their regulation by both endogenous developmental programmes and external triggers (De Veylder *et al.*, 2007). However, when viewed from the context of the whole organism, there are still major unresolved questions as to how cell division processes are integrated into growth and morphogenesis at the organ level (Tsukaya, 2006). For example, although altered patterns of cell division are frequently associated with altered growth, and a causal link has often been inferred from observed changes in constituent cell size and patterning (Autran *et al.*, 2002; Dewitte *et al.*, 2003; Kuwabara *et al.*, 2011), there are many examples where altered cell size/patterning correlates poorly with the resulting growth of the organ (Hemerly *et al.*, 1995; Smith *et al.*, 1996; Horiguchi *et al.*, 2006). As direct comparisons of these investigations are often difficult, due to either different species being investigated or different methods being used to manipulate gene expression, understanding the system-level integration and causal linkage of cell division and leaf growth remains a challenge (Fleming, 2006). One possibility, which has been little considered, is that the altered division pattern and size resulting from manipulating cell-cycle genes has a significant effect on leaf physiology, and it is this flexibility in the linkage of cell division pattern, physiology and growth that accounts for some of the disparate observations that have been made. To test this hypothesis, we describe here a series of experiments in which we have used combined microCT imaging and fluorescence/gas exchange analysis to investigate the physiological outcome of suppression of a key cell-cycle regulator, retinoblastoma-related protein (RBR).

Retinoblastoma protein (Rb) was first identified as a tumour repressor in mammalian systems (Lee *et al.*, 1987; Harbour and Dean, 2000). Since then it has emerged as a central player in the eukaryotic cell cycle, with homologues present in a wide variety of multicellular organisms, including plants (Gutzat *et al.*, 2012). These plant homologues have been termed RBRs, and they share a number of conserved features with other retinoblastoma proteins. An essential function of RBR is to repress E2F transcription factors whose activity is required for progress from the G_1_ to the S phase of the cell cycle. RBR binding to E2Fs is regulated by cyclin-dependent kinases (CDKs), with increased CDK activity leading to hyperphosphorylation of RBR. This leads to release of E2Fs from RBR, allowing progression through the cell cycle. CDK activity itself is subject to control by cyclin proteins, whose expression is regulated by a number of developmental and environmental factors, enabling linkage of cell division to an endogenous programme and modulation of this programme by external growth conditions (Menges *et al.*, 2005; De Veylder *et al.*, 2007; Gutzat *et al.*, 2012).

In addition to a function in repressing progress through the cell cycle, RBR has been implicated in the epigenetic regulation of gene expression. In mammalian systems, Rb has been shown to interact with polycomb proteins, which are known to influence chromatin structure and thus gene expression (Qian *et al.*, 1993; Kotake *et al.*, 2007). The situation is less clear in plants, but there is accumulating evidence that RBR influences histone methylation and thus global patterns of gene expression in a relatively stable fashion (Ach *et al.*, 1997; Jullien *et al.*, 2008; Gutzat *et al.*, 2012). Overall, these data suggest that, in addition to an immediate effect on cell-cycle progression, RBR may have longer-term effects on gene expression, although the precise nature and significance of these effects remains unclear. Among these targets are genes involved in aspects of basic metabolism. For example, RBR has recently been implicated in the transition from heterotrophic to autotrophic growth in seedlings (Gutzat *et al.*, 2011), and such downstream targets may also influence longer-term aspects of plant growth. Finally, RBR may also interact directly with transcription factors involved in asymmetric divisions in the root, indicating that RBR may have a relatively direct effect on cell division patterns, which are likely to influence subsequent cell fate in plant tissues (Cruz-Ramírez *et al*., 2012; Weimer *et al.*, 2012). Thus, an emerging picture is that, in addition to a canonical role in cell-cycle progression, RBR and related proteins may influence cell division and growth processes via other routes.

A number of investigations have been performed regarding the outcome of altered RBR expression and associated cell division changes at the level of the whole organism, mainly using the model genetic organism Arabidopsis (Park *et al.*, 2005; Wildwater *et al.*, 2005; Desvoyes *et al.*, 2006; Borghi *et al.*, 2010; Gutzat *et al.*, 2011; Wachsman *et al.*, 2011). As knockout mutations of the single RBR gene present in Arabidopsis are lethal (Ebel *et al.*, 2004), these experiments utilized a variety of approaches to repress/knockout RBR expression during various stages of development. The experiments have generally shown that loss of RBR leads to promotion of cell proliferation and loss of differentiation, consistent with the idea of the cyclin/CDK/RBR module as a cell-cycle/differentiation decision point which must be integrated into the development of the whole organ (Gutierrez, 2005). With respect to the leaf, the changes observed include a general decrease in cell size and an overall decrease in organ size, i.e. formation of smaller leaves containing a relatively high number of small cells (Borghi *et al.*, 2010). This growth repression following suppression of RBR expression may simply reflect a relationship between RBR and cell size control. Indeed, a recent study suggested that RBR repression leads to cell division occurring at smaller cell size, so that, although cell proliferation occurs as a result of RBR suppression, the increase in the number of cells in the whole organ cannot compensate for the reduction in mean cell size (Kuwabara *et al.*, 2011). However, the altered pattern of cell proliferation/differentiation resulting from RBR suppression also leads to an abnormal histology, and it is possible that this leads to an impaired physiology that limits organ growth.

A classical angiosperm leaf comprises tissues that are specialized for photosynthesis and gas exchange (palisade and spongy mesophyll), which lie adjacent to each other and are bounded by a lower and upper epidermis. These epidermal tissues contain controllable pores (stomata) that allow both CO_2_ uptake and transpiration of water. The leaf contains vessels (phloem and xylem) that are specialized for the transport of materials into and out of the leaf. Each of these tissues consists of cells that are recognizable both by their relative position and their characteristic shape and size. This distinctive cellular architecture arises by co-ordination of growth and cell division in time and space as proliferating cells in the initial leaf primordium gradually undergo the programmed events of differentiation (Fleming, 2005; Braybrook and Kuhlemeier, 2010). A body of literature (experimental and theoretical) suggests that the relative amount of air space and solid tissue, as well as the pattern of air space distribution, has a significant effect on physiological performance, most notably photosynthesis (Parkhurst, 1994; Terashima *et al.*, 2011). Indeed, the robust reproducible process of leaf differentiation suggests that the specific cell forms observed are intimately required for proper functioning of the leaf, and that abnormalities at this supracellular level as a result of changes at the cellular scale will affect the ability of the leaf to acquire CO_2_, for example, and thus have a negative effect on leaf physiology and growth.

We wished to investigate the physiological outcome of altered RBR gene expression. Cell division during leaf development in Arabidopsis follows a predictable trajectory (Donnelly *et al.*, 1999; Kuwabara *et al.*, 2011). Although the precise division pattern of any individual cell is generally difficult to predict, the overall pattern of cell division is reproducible. In particular, there is a phase when cell division terminates throughout the leaf and subsequent growth of the organ predominantly occurs by cell-division independent expansion. We aimed to transiently repress RBR expression during this phase so that cell division was altered during a relatively short time frame, allowing cell growth to recover from RBR suppression. Due to the nature of plant cell division, daughter cells are essentially glued together via shared cell walls and cannot move relative to one another. Thus, although cell growth may recover after disruption (as indicated in previous work, Borghi *et al.*, 2010), any abnormal cell division patterns resulting from RBR suppression are fixed within the developing leaf, allowing assessment of leaf physiology and growth at a time point and developmental stage that is well separated from the time at which RBR manipulation was performed. In addition, as physiological analyses may be extremely sensitive to environmental disturbance, and our strategy involved local supply of an exogenous chemical to stimulate transgene expression, the long temporal separation of gene induction and plant analysis decreased the possibility of the induction procedure itself affecting the results obtained.

In addition to use of combined fluorescence/gas exchange analysis for physiological analysis of the plants (Sharkey *et al.*, 2007), we used micro X–ray computed tomography (microCT) analysis to capture data on the altered cellular architecture resulting from repression of RBR expression. MicroCT allows 3D imaging of objects in an essentially non-invasive fashion (Kaminuma *et al.*, 2008; Dhondt *et al.*, 2010; Tracy *et al.*, 2010), and recent advances in this technology have led to a significant improvement in both the resolution and speed of data capture, extending its potential applications for plant sciences (Pajor *et al.*, 2013). As our results show, this approach enabled a quantitative analysis of the morphological and histological outcome of altered RBR expression that may be related to leaf physiology, and, moreover, allowed us to characterize an unanticipated facet of RBR function in leaves – the control of mesophyll differentiation.

## Results

### Transient suppression of RBR leads to a long-term alteration in leaf form

**Figure 1:**
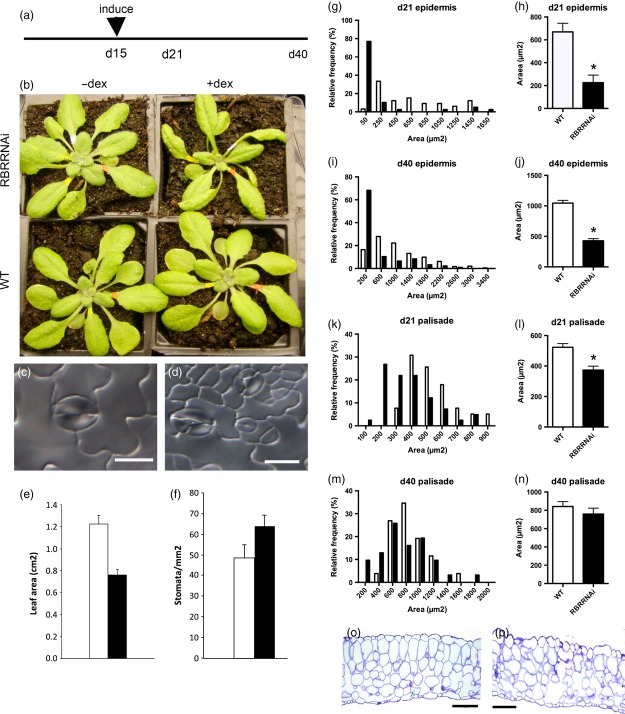
Transient suppression of RBR1 during early development leads to an altered phenotype in the mature leaf. (a) Schematic diagram showing the time course of induction by dexamethasone (DEX) and observation of the plant phenotype. (b) Overview of the rosettes of four plants (RBRRNAi or WT, with or without DEX as indicated) after 40 days of growth. Leaf 8 is marked with red dye at the base of the petiole. (c,d) Images of stomata from mock-induced (c) and induced (d) RBRRNAi plants. (e, f) Comparison of the leaf 8 final area (e) and stomatal density (f) in WT plants (open bars) and induced RBRRNAi plants (closed bars). Values are means and SD (*n* ≥ 6 for each treatment). (g–n) Comparison of epidermal cell area at 21 DAS (g, h) and 40 DAS (i, j), and palisade cell area at 21 DAS (k, l) and 40 DAS (m, n) in leaf 8 of mock-induced WT (open bars) and induced RBRRNAi plants (closed bars). (g), (i), (k) and (m) show the relative frequency of cell size for the treatments; (h), (j), (l) and (n) show the mean values of cell area for the treatments. Error bars indicate SEM (*n *>* *30). Asterisks indicate statistically significant differences compared with WT (*P *<* *0.01) (Wilcoxon rank test). (o, p) Cross-sections from mock-induced (o) and induced (p) RBRRNAi plants showing abnormal cellular architecture. Scale bars = 1 cm (b), 20 μm (c,d) and 100 μm (o,p).

To establish a transient induction system, we utilized previously characterized transgenic lines of Arabidopsis containing a pOpOFF::RBR construct (Kuwabara *et al.*, 2011). In these lines (hereafter termed RBRRNAi), exogenous supply of the inducer dexamethasome (DEX) leads to transcription of an RBRRNAi cassette and decreased levels of both RBR transcript and protein. Our previous data indicated that a decrease in transcript level occurred within 24 h of induction, with decreased protein accumulation being detectable after 48–72 h (Kuwabara *et al.*, 2011). We germinated seedlings on soil, and transplanted plants with four leaves to individual pots. At 15 days after sowing (DAS), seedlings with six visible leaves were treated once on the apex with a drop (30 μl) of DEX inducer, then the seedlings were allowed to grow for a further 25 days to maturity (Figure1a). A series of growth analyses indicated that, at 15 DAS, leaf number 8 had a length of 300–600 μm, and that termination of cell division is just starting within the leaf at this developmental stage (Kuwabara *et al.*, 2011). As this leaf grew out, it was marked at the petiole to aid identification, and the final size of the leaf was measured. When the leaf had reached its final size (at 40 DAS, Figure S1), we performed fluorescence and gas exchange measurements to obtain a set of physiological measurements, followed by microCT analysis to obtain a set of morphological parameters.

By the end of the experiment, leaf 8 in both induced RBRRNAi and WT control plants had grown several hundred-fold in length following the induction process, but a clear decrease in final leaf area was observed after transient RBR suppression (Figure1b,e). Analysis of the leaf epidermis indicated accumulation of smaller cells, including the occurrence of aberrant stomata (Figure1c,d) and an increase in stomatal density (Figure1f) in DEX-treated RBRRNAi plants. With respect to the palisade mesophyll, there was no significant difference in mean cell area at the end of the experiment (Figure1n), although there was a tendency for smaller cells to be present in the induced RBRRNAi leaves (Figure1m). When a similar analysis was performed at 21 DAS (i.e. 6 days after induction, Figure1a), the palisade cells in the induced RBRRNAi leaves showed an accumulation of smaller cells that were not apparent in controls (Figure1k,l), and this difference was significant at the 0.01% confidence limit by non-parametric analysis. With respect to the epidermal cell phenotype, quantitative analysis of the cell area distribution indicated that the induced RBRRNAi leaves contained a higher relative frequency of smaller cells than observed in control leaves at both 21 and 40 DAS (Figure1g–j). Statistical analysis (non-parametric test) indicated that the distribution of areas of epidermal cells in the induced RBRRNAi leaves was significantly different from that in controls at both 21 and 40 DAS.

**Figure 2:**
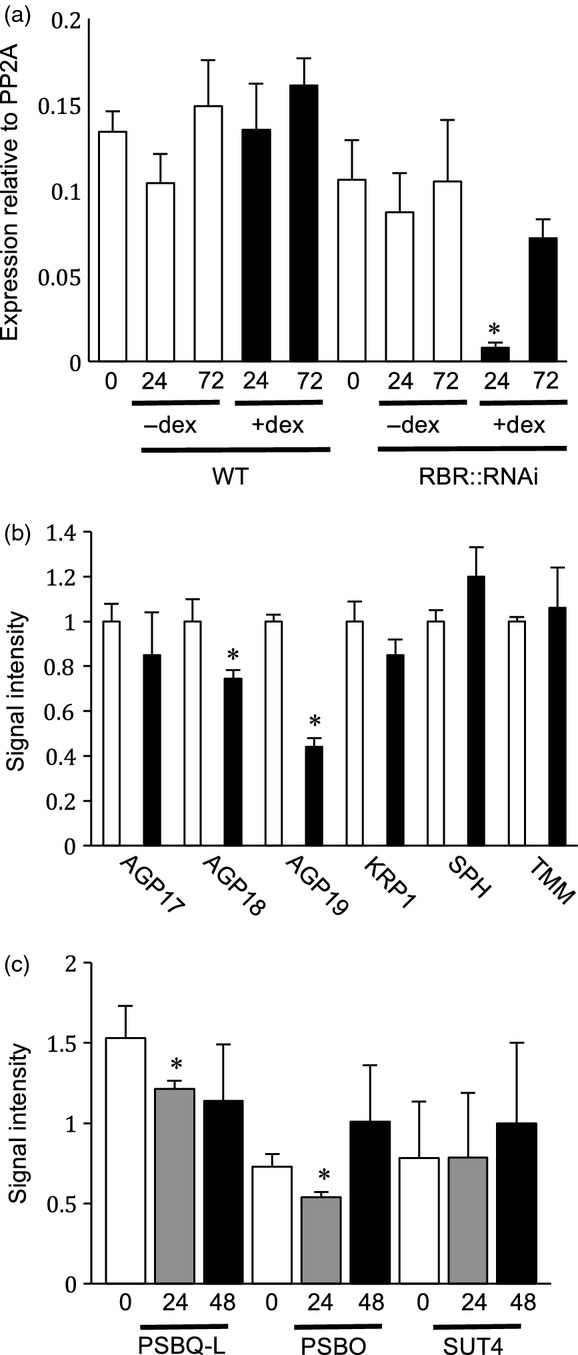
Quantitative RT–PCR analysis of RBR1 and AGP19 transcript levels. (a) Levels of *RBR1* transcript relative to the control *PP2A* gene at 24 and 72 h after treatment at 15 DAS with or without 5 μm DEX in either WT or RBRRNAi seedlings. Error bars indicate SEM (*n *=* *3). (b) Relative transcript levels of a series of genes (as indicated) in 17-day-old RBRRNAi seedlings either without treatment (open bars) or after treatment with 5 μm DEX for 48 h (closed bars). Levels are normalized to the initial level of the relevant transcript of the mock-induced plants for each gene. Error bars indicate SEM (*n *=* *3). (c) Transcript levels of genes involved in metabolism (*PSBQ–L*, *PSBO* and *SUT4*: Gutzat *et al.*, 2011) at various time points (0, 24 and 48 h) after induction of RBRRNAi seedlings (15 days old) with 5 μm DEX. Values are relative to expression of the control *PP2A* gene. Asterisks indicate statistically significant differences (*P *<* *0.05) relative to relevant *t *=* *0 h sample within a–c. Error bars indicate SEM (*n *=* *3).

The epidermal and growth phenotypes are consistent with those reported previously subsequent to more prolonged suppression of RBR (Fleming, 2005; Park *et al.*, 2005; Borghi *et al.*, 2010; Kuwabara *et al.*, 2011), indicating that the brief induction procedure was sufficient to induce changes in cellular architecture that were fixed in the mature leaf. To confirm the transient nature of the induction, we performed a quantitative PCR analysis of leaves after DEX induction to estimate the RBR transcript level. These results (Figure2a) indicated that there was a significant (*P *<* *0.05) decrease in RBR transcript level within 24 h of induction. By 72 h, the RBR transcript level was recovering and was not statistically different from that in control plants. Previous analysis of these lines demonstrated a decrease in RBR protein level following prolonged suppression of RBR expression (Kuwabara *et al.*, 2011). The limiting amounts of sample target tissue used here and the transient nature of the transcriptional response precluded analysis of the RBR protein level. As a further test for the transient nature of the induction process, we exploited the fact that the construct used (pOpOFF::RBR) contains a bi-directional promoter, such that expression of a GUS reporter gene may be used as a proxy for expression of the RBRRNAi sequence used to suppress RBR expression (Wielopolska *et al.*, 2005). Within 24 h of DEX treatment of the 15 DAS plants, a strong GUS signal was observed in the target leaf and adjacent leaves (Figure S2A,B) but no GUS signal was apparent in the target leaves after 5 days (Figure S2C). In addition, we performed a quantitative PCR analysis of selected lead genes linked with metabolism, as a previous study had highlighted these as early markers for RBR gene suppression. As shown in Figure2(c), two of these marker genes (PSBQ–L and PSBO, extrinsic sub-units of photosystem II) showed a decrease in the transcript level 24 h after suppression of RBR, consistent with previous data (Gutzat *et al.*, 2011).

Taken together, the data from both this and our previous work indicate that our method led to transient suppression of RBR in the target tissue, and that this transient suppression was sufficient to induce a phenotype (at the level of overall leaf growth and cell size) that was very similar to that described in experiments in which RBR was repressed over a much longer time period. Although a long-term change in epidermal cell patterning was induced, the altered cell division in the palisade mesophyll was more transient. In addition, histological analysis of the mature leaves suggested an alteration in mesophyll cell packing after suppression of RBR (Figure1o,p), a phenotype that had not been previously reported. To further investigate this change in leaf cellular architecture in more detail, we used a microCT scanning approach.

### Suppression of RBR leads to altered mesophyll porosity

**Figure 3:**
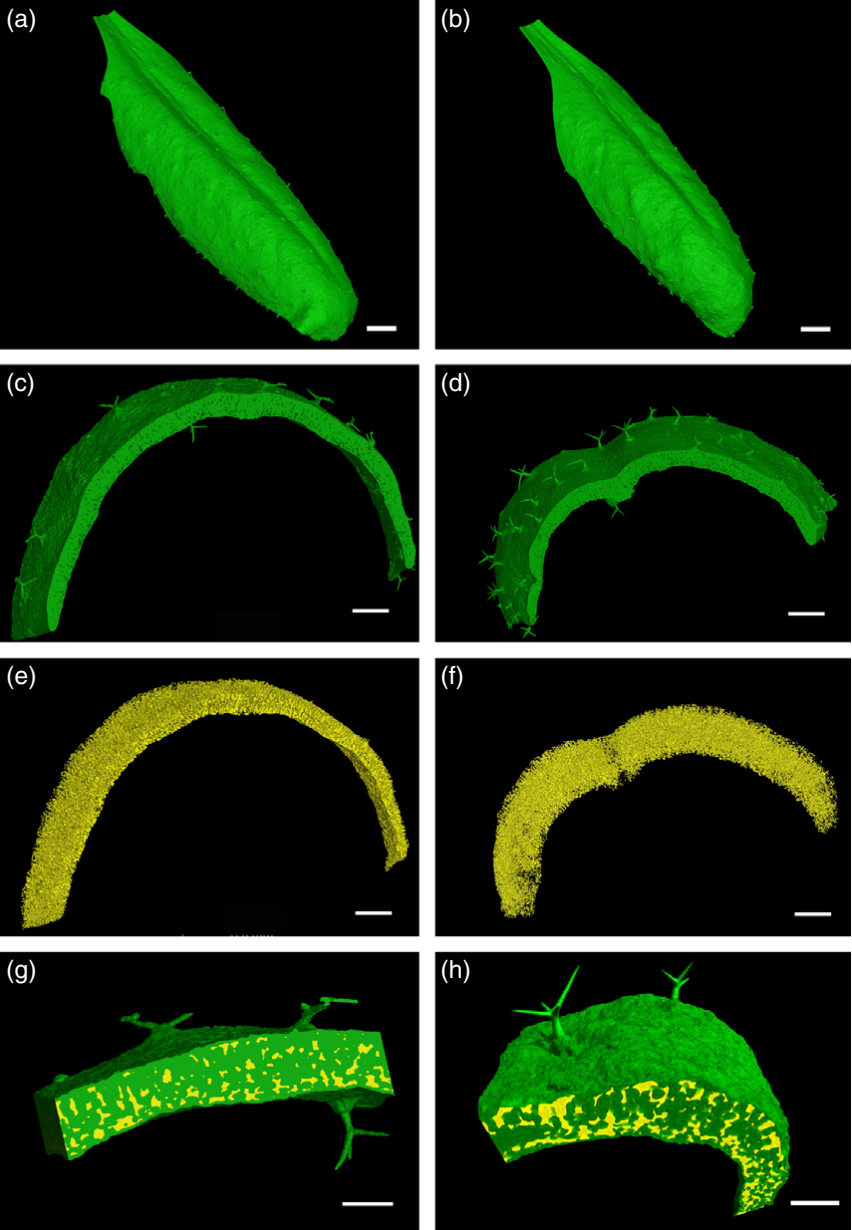
MicroCT analysis of Arabidopsis leaves. (a, b) Control WT (a) and induced RBRRNAi (b) leaves after 40 days growth. (c, d) Sections showing solid tissue extracted from images in (a) and (b). (e, f) Sections showing air space extracted from images in (a) and (b). (g, h) Sections showing combined solid tissue (green) and air space (yellow), to provide a visual impression of the air space distribution in sections of either WT (g) or induced RBRRNAi (h) leaves. Scale bar = 2 mm (a,b), 500 μm (c–f) and 250 μm (g, h).

MicroCT scanning was used to produce 3D images of leaves (Figure3a,b). As the various components of an organ have different X–ray attenuation coefficients, the process allows discrimination between liquid-filled cells and the air-filled intercellular space of the leaf. Thus scans of either control (Figure3a) or induced RBRRNAi leaves (Figure3b) may be separated into those parts representing solid plant tissue (green in Figure3c,d) and the air spaces within the tissue (yellow in Figure3e,f). Combining the colour-coded solid and air spaces provides a visual impression of the relative amounts and distribution of the two volumes (Figure3g,h). These images may be explored as 3D re-constructions and movies (e.g. Movie S1), and, more importantly, the data may be subjected to quantitative analyses, as described below.

**Figure 4:**
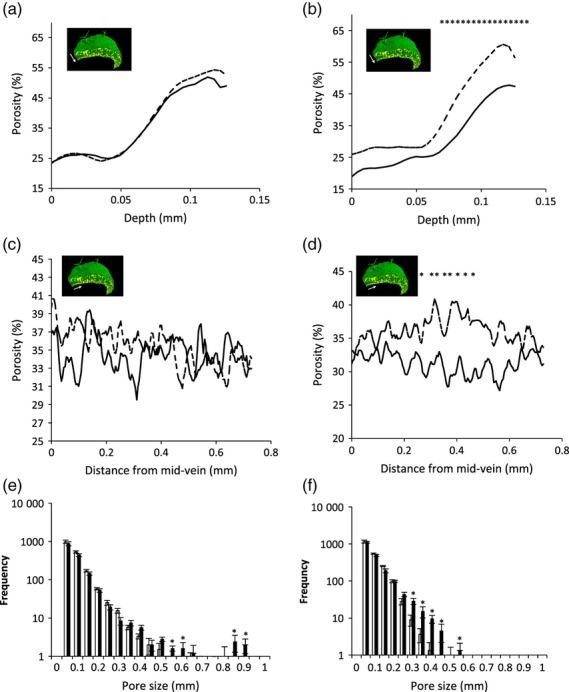
Quantitative analysis of WT and RBR-suppressed leaves after microCT imaging. (a) Porosity along the vertical axis (adaxial to abaxial surface, as indicated in the inset) for WT leaves either treated (dashed line) or not treated (solid line) with DEX at 15 DAS and analysed at 40 DAS. For clarity, the variance is not shown, but there was no significant difference in porosity at any point along the adaxial/abaxial axis. (b) Porosity along the vertical axis (adaxial to abaxial surface, as indicated in the inset) for RBRRNAi leaves either induced (dashed line) or non-induced (solid line) with DEX at 15 DAS and analysed at 40 DAS. For clarity, the variance is not shown, but a statistically significant difference in porosity (*P *<* *0.05, Student's *t* test) was observed in the lower, abaxial part of the leaf (indicated by asterisks). (c) Porosity along the horizontal axis (mid-vein towards margin, as indicated in the inset) for WT leaves either treated (dashed line) or not treated (solid line) with DEX at 15 DAS and analysed at 40 DAS. For clarity, the variance is not shown, but there was no significant difference in the porosity value at any point along the adaxial/abaxial axis. (d) Porosity along the horizontal axis (mid-vein towards margin, as indicated in the inset) for RBRRNAi leaves either treated (dashed) or not treated (solid line) with DEX at 15 DAS and analysed at 40 DAS. For clarity, the variance is not shown, but a statistically significant difference in porosity (*P *<* *0.05, Student's *t* test) was calculated in a region approximately 0.25–0.45 mm from the mid-vein (as indicated by asterisks). (e) Pore size frequency distribution for WT leaves (open columns) and induced RBRRNAi leaves (filled columns) along the vertical axis (adaxial/abaxial). Asterisks indicate pore sizes for which there is a statistically significant difference in frequency (*P *<* *0.05, Student's *t* test) between the two treatments. Error bars represent SD (*n *=* *6). (f) Pore size frequency distribution for WT leaves (open columns) and induced RBRRNAi leaves (filled columns) along the horizontal axis (mid-vein towards margin). Asterisks indicate pore sizes for which there is a statistically significant difference in frequency(*P *<* *0.05, Student's *t* test) between the two treatments. Error bars represent SD (*n *=* *6).

Figure4 shows an analysis of the porosity (relative amount of air space to total volume) of leaf 8 for both WT plants (treated and non-treated) and RBRRNAi plants (induced and non-induced) derived from microCT analyses of seven samples per treatment. Considering first the porosity along the vertical axis from the leaf adaxial surface to the abaxial surface, there was a small peak just below the adaxial surface for treated and non-treated WT leaves (Figure4a), but the overall trend was for increasing porosity towards the abaxial surface, with no significant difference between induced and non-induced leaves. For the RBRRNAi leaves, the porosity along the vertical axis was generally higher for the treated leaves than for non-treated leaves, and this difference was statistically significant (*P *<* *0.05) in the abaxial region of the leaf (where spongy mesophyll differentiation normally occurs) (Figure4b). When the porosity along the horizontal axis (across the leaf lamina from the mid-vein towards the margin) was analysed, there was no significant difference between treated and non-treated WT leaves as the distance from the mid-vein increased (Figure4c). However, analysis of the RBRRNAi samples indicated a significant difference (*P *<* *0.05) between the treated and non-treated samples in a region distant from the mid-vein, with the induced RBRRNAi leaves having an increased porosity (Figure4d). The porosity analysis gives a measure of the relative amount of air space to the total volume of the leaf. The datasets may also be interrogated for mean pore size, which indicates how this air space is divided within the tissue. This analysis revealed that there was an increase in relatively large pores (> 0.5 mm for the vertical axis and > 0.3 mm for the horizontal axis) in the induced RBRRNAi leaves compared with WT (Figure4e,f).

MicroCT analysis of earlier developmental stages of leaf 8 revealed a gradual increase in porosity over time. As shown in Figure S3, at 28 and 35 DAS (16 and 23 days post-induction), the pattern of porosity across the adaxial/abaxial axis of the young leaf was similar to that observed in the mature leaf (40 DAS), but the absolute levels of porosity were lower. For example, the upper mesophyll cells had a mean porosity at 28 DAS of < 15%, whereas this had increased to almost 25% by 40 DAS. Analysis of leaf 8 at 21 DAS (6 days post-induction) revealed virtually no air space under any conditions, and thus very low porosity throughout the leaf, but our analysis of the cell division pattern (Figure1) indicated clear changes in the induced RBRRNAi plants by this time point. Thus, the change in air space distribution (porosity) occurred at some time point after the change in cell division pattern.

### RBR suppression leads to altered leaf stomatal conductance and ΦPSII

**Figure 5:**
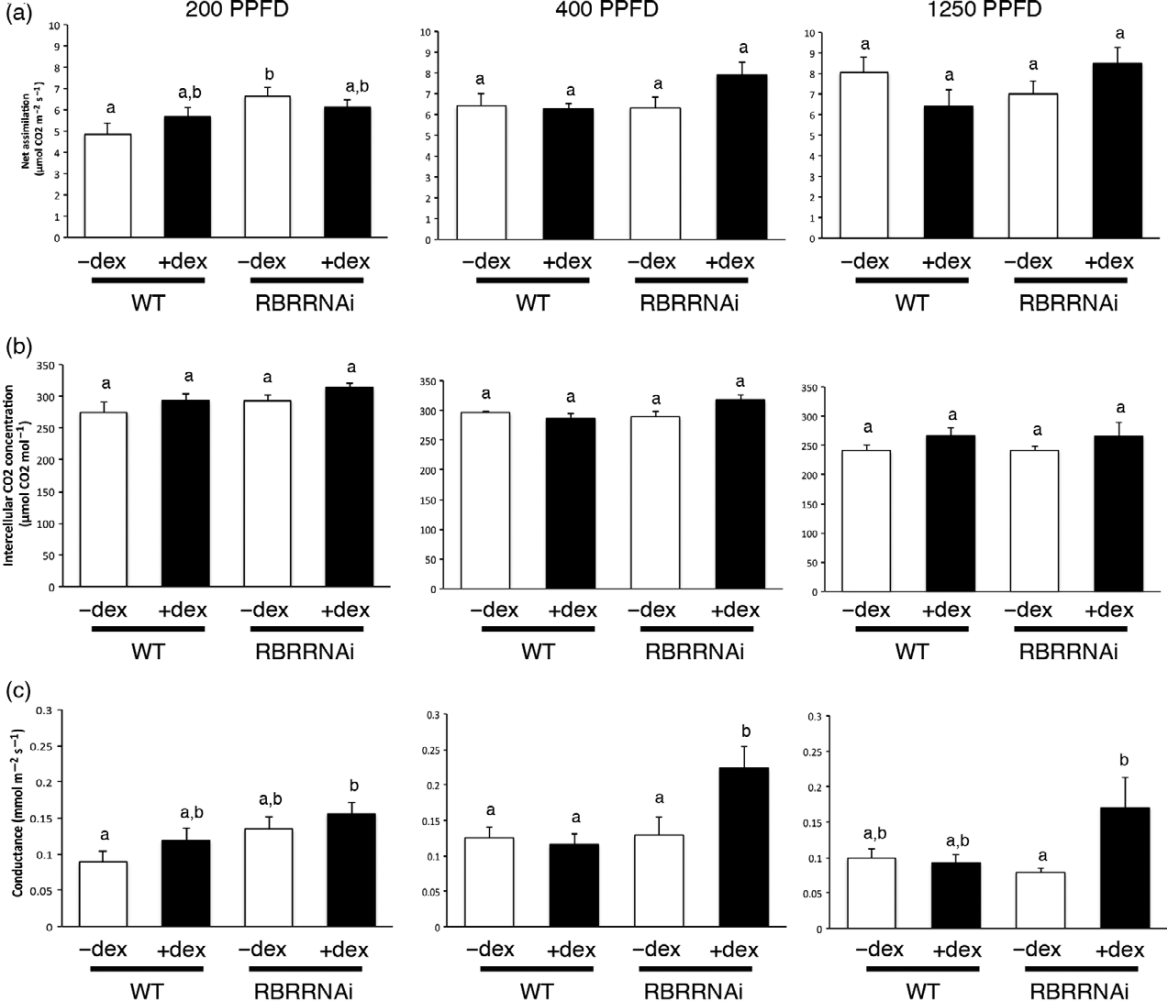
Physiological analysis of RBR-suppressed leaves. (a) Net assimilation rate, (b) intercellular CO_2_ concentration and (c) stomatal conductance for water for WT or RBRRNAi plants either treated (+DEX) or not treated (−DEX) at 15 DAS and analysed at 40 DAS under three irradiances (200, 400 and 1250 μmol m^−2^ sec^−1^ photosynthetic photon flux density (PPFD), as indicated). Error bars indicate one standard error of the mean (*n *>* *8 for each treatment). Each set of data was analysed by anova and a *post hoc* Tukey test to compare samples. Bars with identical letters are not significantly different from each other at a confidence limit of 0.05.

The analysis of leaf structure indicated a number of alterations in cellular architecture that may be expected to affect leaf physiology. For example, stomatal pores are key to controlling both the influx of CO_2_ for photosynthesis and the loss of water via transpiration. The presence of abnormal stomata and altered density may be expected to alter leaf gas exchange. The distribution of intercellular air space may also affect the flux of gases within the leaf and influence the photosynthetic performance of the leaf (Parkhurst, 1994; Terashima *et al.*, 2011). Finally, the abnormal cellular patterning observed in the epidermis and other layers may also affect the ability of the leaf to capture and retain light energy, potentially having a deleterious effect on photosynthetic performance and thus growth. To investigate whether these altered cellular parameters actually influence the leaf's physiological performance, we performed a series of combined chlorophyll fluorescence/gas exchange analyses at a range of irradiances to test the performance of the RBR-suppressed leaves (and controls) under different environmental conditions (Figure5).

Analysis of leaf stomatal conductance (Figure5c) indicated that, under standard growth irradiance (200 μmol m^−2^ sec^−1^), the induced RBRRNAi leaves had a slightly increased conductance compared with both non-induced RBRRNAi leaves and WT treated and non-treated leaves. When the irradiance was increased (400 μmol m^−2^ sec^−1^) this difference in conductance became significant (*P *<* *0.05), with the induced RBRRNAi leaves showing a higher conductance. Under very high irradiance levels (1250 μmol m^−2^ sec^−1^), the induced RBRRNAi leaves showed a higher conductance compared with non-induced RBRRNAi leaves, but the variance was such that this difference was not significant compared with WT leaves. Altered stomatal function and altered tissue porosity and pore size may be expected to alter the flux of gases into and within a leaf and thus influence intercellular CO_2_ concentration. Although a tendency for increased intercellular CO_2_ concentration was observed with the DEX-treated RBRRNAi leaves (Figure5b), this was not statistically significant in any individual comparison. When the photosynthetic assimilation rates of the samples under different irradiances were compared, there was no evidence of a deleterious effect on CO_2_ assimilation following suppression of RBR, with the induced RBR leaves showing mean assimilation rates comparable to those measured in the other treatments (Figure5a).

**Figure 6:**
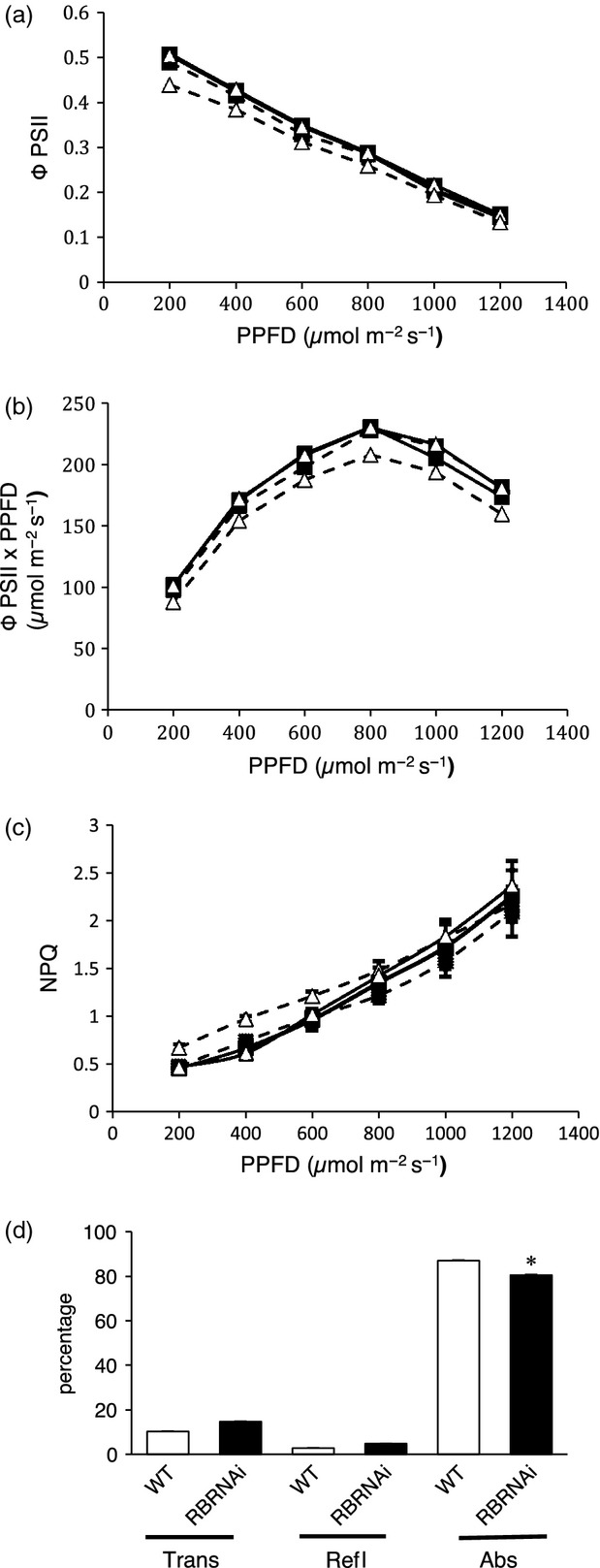
Photosynthetic performance and light absorption by leaves. (a) ΦPSII at various irradiances for WT leaves (filled squares) and RBRRNAi leaves (open triangles) after either DEX treatment (dashed lines) at 15 DAS and analysis at 40 DAS, or without treatment (solid lines). Error bars (where visible) indicate one standard error of the mean (*n *>* *6). (b) ΦPSII × incident irradiance at various irradiances for WT leaves (filled squares) and RBRRNAi leaves (open triangles) after either DEX treatment (dashed lines) at 15 DAS and analysis at 40 DAS, or without treatment (solid lines). Error bars (where visible) indicate one standard error of the mean (*n *>* *6). (c) Non-photochemical quenching at various irradiances for WT leaves (filled squares) and RBRRNAi leaves (open triangles) after either DEX treatment (dashed lines) at 15 DAS and analysis at 40 DAS, or without treatment (solid lines). Error bars (where visible) indicate one standard error of the mean (*n *>* *6). (d) Percentage white light transmission, reflection and calculated absorbance for WT and RBRRNAi leaves treated with DEX at 15 DAS and analysed at 40 DAS. Error bars (where visible) indicate one standard error of the mean (*n *>* *6).

Analysis of the integrity of the photosynthetic apparatus revealed no difference between the treatments and genotypes, with an *F*_v_/*F*_m_ value of approximately 0.8 in all treatments (as also suggested by maintenance of the carbon assimilation rates). However, the ΦPSII values [a measure of the proportion of light absorbed by chlorophyll associated with photosystem II (PSII) that is used for photochemistry; Maxwell and Johnson, 2000; ] were decreased in the RBR-suppressed leaves at all irradiances from 200 to 1200 μmol m^−2^ sec^−1^ (Figure6a). When the different incident irradiance on the leaves was taken into account, this difference was even more marked, with the induced RBRRNAi leaves showing a significantly (*P *<* *0.05) lower efficiency of electron transport through photosystem II (Figure6b). Conversely, the induced RBRRNAi leaves showed higher values for non-photochemical quenching associated with absorbed light not used for photochemistry at photosynthetically active photon flux densities of 600 μmol m^−2^ sec^−1^ and below (Figure6c). These data suggested that the RBR-suppressed leaves dissipated a greater proportion of incident light energy as heat, and used a reduced proportion in photochemistry, which may reflect an altered underlying physiological mechanism or be related to an altered cellular architecture influencing light absorption. To investigate whether the changes in cellular architecture in the induced RBRRNAi leaves altered light absorption, we measured this parameter in induced and control leaves. The results indicated that the induced RBRRNAi leaves had a higher reflectance and higher transmission of light, leading to overall significantly (*P *<* *0.05) lower percentage of light absorbance, with a mean total light absorption of 86% in control leaves compared with 80% in the induced RBRRNAi leaves (Figure6d). Total white light absorption in the RBR-suppressed leaves was therefore approximately 93% that of control leaves. Despite this decrease in relative light absorption and ΦPSII level, the assimilation rates in the induced RBRRNAi leaves did not differ from the controls. To test whether this was due to a change in gross biochemical capacity for light absorption, we measured bulk chlorophyll levels in the induced RBRRNAi and control leaves. These results indicated no significant difference in chlorophyll level per tissue fresh weight in the induced RBRRNAi leaves relative to controls (Figure S4). Analysis of anthocyanin content (an indicator of the light stress response) also did not reveal any significant difference between samples in response to high irradiance (Figure S4).

### Stomatal-targeted suppression of RBR does not lead to altered leaf porosity

**Figure 7:**
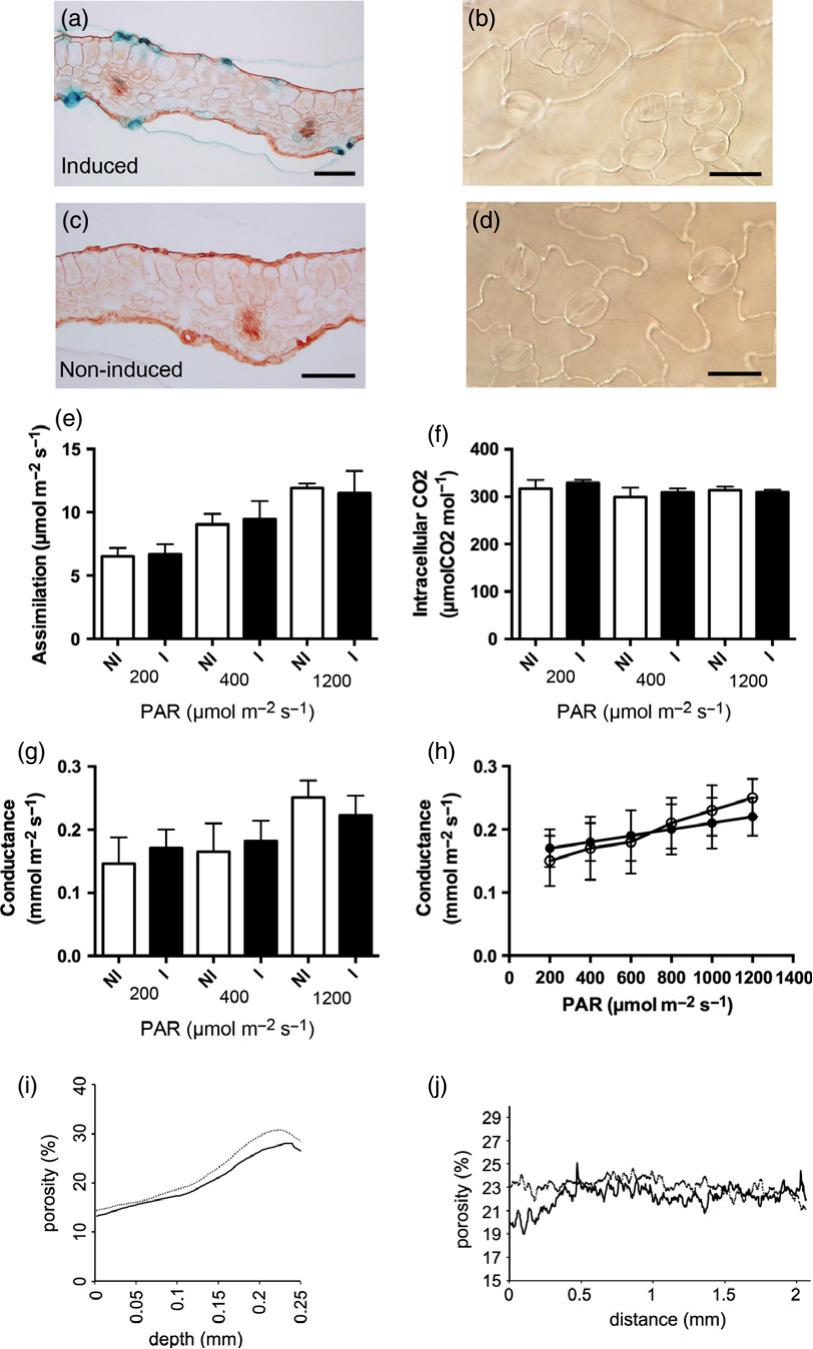
Combined light microscopy, microCT and physiological analysis of pFAMA::RBRRNAi leaves. (a) GUS histochemistry of a transverse section of a leaf from a pFAMA::RBRRNAi plant 6 days post-induction. Signal (blue) is observed in stomata. Scale bar = 200 μm. (b) Nomarski image of a region of the epidermis of a leaf from a pFAMA::RBRRNAi plant 6 days post-induction showing abnormal stomata. Scale bar = 20 μm. (c) As (a) but for a non-induced leaf. Scale bar = 200 μm. (d) As (b) but for a non-induced leaf. Scale bar = 20 μm. (e) Net assimilation rate, (f) Intercellular CO_2_ concentration and (g) stomatal conductance of leaves of pFAMA::RBRRNAi plants induced (solid bars) or not induced (open bars) at 15 DAS and analysed at 40 DAS under three irradiances (200, 400 and 1200 μmol m^−2^ sec^−1^). Error bars indicate SD (*n *=* *6 for each treatment). (h) Stomatal conductance of leaves of pFAMA::RBRRNAi plants over a range of (Photosynthetically Active Radiation, PAR) showing the trend of change in conductance for leaves induced (solid symbols) or not induced (open symbols) at 15 DAS prior to analysis at 40 DAS. Error bars indicate SD (*n *=* *6). Regression analysis combined with anova indicated that the gradients of the two lines are significantly different (*P *<* *0.05). (i,j) Porosity along the vertical axis (adaxial to abaxial surface) (i) and horizontal axis (j) of leaf 8 of pFAMA::RBRRNAi plants either treated (dashed line) or not treated (solid line) with DEX at 15 DAS and analysed at 40 DAS. For clarity, the variance is not shown, but there was no significant difference in porosity value at any point along the vertical or horizontal axis between induced and non-induced leaves.

Induction of the RBRRNAi leaves leads to suppression of RBR in all tissues, raising the possibility that some of the observed effects may be indirect. For example, altered stomatal differentiation may lead to altered gas flux, which may affect mesophyll differentiation. To test this possibility, we created transgenic plants in which expression of the RBRRNAi construct was under the control of the inducible pFAMA promoter, which may be used to target gene expression to differentiating guard cells (Ohashi-Ito and Bergmann, 2006). As shown in Figure7(a), induction of pFAMA::RBRRNAi led to GUS reporter gene expression in guard cells, which was not observed in non-induced leaves (Figure7c). Analysis of the induced leaves revealed the presence of abnormal stomata and stomatal clusters (compare Figure7b and Figure7d). When the induced and non-induced mature leaves were analysed for their ability to assimilate carbon dioxide (Figure7e) and their intercellular CO_2_ concentration (Figure7f), no significant different was observed under any of the three irradiance levels tested, comparable to the results observed in the induced RBRRNAi leaves. Analysis of stomatal conductance also did not reveal any significant difference between induced and non-induced leaves at any irradiance level (Figure7g). However, when the change in stomatal conductance was compared over a range of irradiance levels (Figure7h), the induced pFAMA::RBRRNAi leaves showed a significantly (*P *<* *0.05) lower rate of change of conductance with decreasing irradiance, suggesting an impairment in the stomatal response to altered irradiance. MicroCT analysis of the pFAMA::RBRRNAi leaves indicated no significant difference in porosity distribution between induced and non-induced leaves along either the vertical axis (Figure7i) or the horizontal axis (Figure7j).

### AGP19 as a potential mediator of RBR control of mesophyll differentiation

Our data indicated that transient suppression of RBR during leaf development led to altered mesophyll differentiation such that a more porous cellular architecture was formed. Despite the accepted role of spongy mesophyll as a key element of the leaf that allows gas exchange both within the leaf and to the external environment via stomata, surprisingly little is known about the molecular control of mesophyll differentiation. Previously, Yang *et al*. (2007) identified altered mesophyll structure as an element of the phenotype of a mutant in the *AtAGP19* gene, which encodes a member of the arabinogalactan protein (AGP) family. AGPs are cell-wall proteins that are encoded by a large gene family, and, although implicated in cell-cell adhesion/communication, our understanding of their role and control of expression remains limited (Seifert and Roberts, 2007; Ellis *et al.*, 2010). Interestingly, a recent microarray analysis of RBR-mediated gene expression identified *AtAGP17* and *AtAGP18* as potential targets for RBR (Borghi *et al.*, 2010). The *AtAGP19* sequence was not on the microarray used in the previous report, so we investigated whether the *AtAGP19* transcript level was altered after suppression of RBR. As shown in Figure2(b), inducible suppression of RBR was followed by a decrease in the transcript level for *AtAGP18* (as previously reported) and, to an even greater extent, for *AtAGP19*. Analysis of transcripts for the control genes *AtSPCH* (*SPEECHLESS*) and *AtTMM* (*TOO MANY MOUTHS*) indicated an increase in their mean level in line with previous reports (Borghi *et al.*, 2010), although these differences were not significantly different under our conditions. These data support the hypothesis that levels of the *AtAGP19* transcript decrease in the young leaf in response to suppression of *RBR1*.

**Figure 8:**
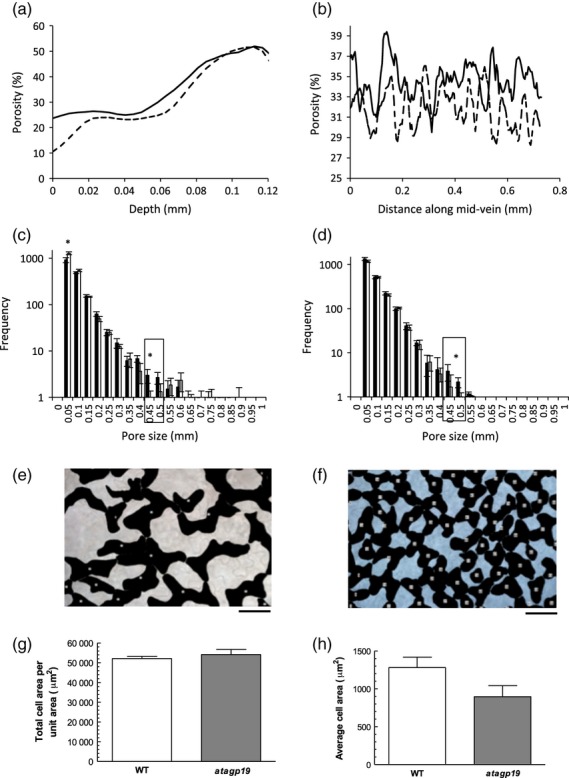
MicroCT analysis of the *atagp19* mutant. (a) Porosity along the vertical axis (adaxial to abaxial surface) for WT leaf 8 (solid line) or *atagp19* mutant leaf 8 (dashed line) at maturity. For clarity, the variance is not shown, but there was no significant difference in porosity value at any point along the adaxial/abaxial axis. (b) Porosity along the horizontal axis (mid-vein towards margin) for WT leaf 8 (solid line) or *atagp19* mutant leaf 8 (dashed line) at maturity. For clarity, the variance is not shown, but there was no significant difference in porosity value at any point along the horizontal axis. (c) Pore size frequency distribution for WT leaves (filled columns) and *atagp19* leaves (open columns) along the vertical axis (adaxial/abaxial). Asterisks indicate a statistically significant difference in pore size frequency (*P *<* *0.05). Error bars indicate SD (*n *=* *6). (d) Pore size frequency distribution for WT leaves (filled columns) and *atagp19* leaves (open columns) along the horizontal axis (mid-vein towards margin). The asterisk indicates a statistically significant difference in pore size frequency (*P *<* *0.05). Error bars indicate SD (*n *=* *6). (e,f) Representative differential interference contrast micrographs of the mesophyll of a WT leaf (e) and an *atagp19* leaf (f). Solid tissue is shown in black. Scale bar = 50 μm. (g) Comparison of total cell area calculated from differential interference contrast micrographs of WT and *atagp19* leaves. (h) Comparison of mean cell area calculated from differential interference contrast micrographs of WT and *atagp19* leaves. Error bars indicate SEM (*n *>* *6). The samples are significantly different at *P *<* *0.05 (Student's *t* test).

To analyse the phenotypic outcome of loss of *AtAGP19* gene expression, we analysed an *atagp19* knockout mutant by microCT. Analysis of porosity along both the vertical axis (adaxial to abaxial surface) (Figure8a) and the horizontal axis (across the leaf from the mid-vein) (Figure8b) revealed no significant difference relative to the WT control. However, when the pore size distribution was analysed, a significant difference along the vertical axis was observed between the *atagp19* mutant and control leaves. In particular, there was a loss of relatively large pores (0.45–0.55 mm) and an increase in the frequency of relatively small pores (<0.05 mm) (Figure8c). A decrease in the frequency of relatively large pores was also measured along the horizontal leaf axis, but no significant change in the frequency of smaller pores was noted (Figure8d). To validate these data, we analysed a series of cleared *atagp19* and wild-type leaves using differential interference contrast microscopy, and manually outlined cells and air spaces. Visual analysis of these images (Figure8e,f) suggested a clear difference between the two genotypes, with the *atagp19* images showing smaller cells and more air spaces. Statistical analysis of these data indicated that the actual relative amount of tissue/air space was not significantly different between the *atagp19* mutant and wild-type tissue (Figure8g), supporting the microCT porosity analysis. However, the mean mesophyll cell size in the *atagp19* mutant was significantly smaller (*P *<* *0.05) than for wild-type tissue (Figure8h).

## Discussion

### Transient suppression of RBR during leaf development leads to a long-term change in leaf form and internal structure

The initial premise for the work reported here was that a transient repression of *RBR1* gene expression during a specific phase of leaf development would be sufficient to alter the patterns of cell division, but that, on recovery of *RBR1* gene expression, the constituent cells would recover their normal growth such that we would be able to determine the relative contribution of cell division pattern on leaf function (physiology) from the more direct influence of altered RBR expression on potentially many aspects of cell function. Our results indicate that a transient suppression of *RBR1* was indeed sufficient to alter the cell division pattern. At the whole-leaf scale, the phenotype observed as a result of this transient RBR repression was very similar to that reported for prolonged suppression of RBR (smaller leaf area, smaller epidermal cells, abnormal stomata) (Park *et al.*, 2005; Borghi *et al.*, 2010). These results indicate that tight regulation of RBR activity is vital during early leaf development.

Our analysis of the leaf internal cellular architecture supported our initial hypothesis that the altered cell division pattern resulting from RBR suppression may lead to an altered porosity that would alter gas exchange and thus at least partially account for the observed growth suppression (Parkhurst, 1994; Terashima *et al.*, 2011). Our results provide a quantification of leaf porosity that was derived from a 3D dataset much more quickly than by classical methods involving interpretation of 2D images (Parkhurst, 1994). The actual values of porosity obtained are comparable to published values for other leaf types, suggesting that the microCT approach provides an accurate estimate of this parameter (as also indicated by our manual validation of the *atagp19* data). The ease with which these 3D datasets may be interrogated opens the door to work in which the variance in porosity in specific regions of a leaf may be compared and related to classical theories of mesophyll conductance and its role in photosynthetic function, for example.

However, analysis of the physiology of the induced RBRRNAi plants indicated that, despite the altered cellular architecture, the leaves were entirely capable of performing the basic function of a leaf, i.e. photosynthesis, with assimilation rates being comparable to those in control samples. Thus, the observed decreased growth of the RBR-suppressed leaves is unlikely to be due to inability of the leaves to obtain sufficient carbon or light energy for growth. The RBR-suppressed leaves had a higher stomatal conductance at a photosynthetically active radiation (PAR) of 400 μmol m^−2^ sec^−1^, consistent with the observed occurrence of abnormal stomata. However, the plants were grown under conditions of relatively high humidity, and decreased leaf growth was also observed in induced RBRRNAi seedlings cultured *in vitro* (Kuwabara *et al.*, 2011) in which the relative humidity approaches saturation. In addition, analysis of leaves in which RBR suppression was targeted to developing stomata (pFAMA::RBRRNAi) did not lead to any significant alteration in mesophyll porosity or change in carbon assimilation, indicating that the increased mesophyll porosity observed in the induced RBRRNAi leaves reflects a direct role of RBR in mesophyll differentiation. Although a change of stomatal conductance was observed in the induced pFAMA::RBRRNAi leaves, this was not as dramatic as that observed in the induced RBRRNAi leaves, suggesting that an interaction or combined effect of RBR suppression in both layers of the leaf may be required for phenotype development.

RBR and related proteins are known to interact with a series of partners linked to long-term regulation of gene expression (Qian *et al.*, 1993; Johnston *et al.*, 2008; Jullien *et al.*, 2008). The precise identity of the eventual target genes is largely unknown, but factors that determine cell size/growth and/or metabolism may be key components leading to the long-term limitation of growth observed after transient suppression of RBR, especially bearing in mind the link between RBR and autotrophic growth (Gutzat *et al.*, 2011). Despite the limited tissue sample size, further investigation of potential early leaf developmental stage-specific RBR epigenetic targets may provide powerful insights into the regulation of leaf growth.

### Cell-wall structure as a potential downstream target of RBR

Our analysis revealed a previously undescribed aspect of RBR function: after transient suppression, partitioning of the leaf volume into air space and solid tissue was altered, leading to increased porosity accompanied by an increase in the frequency of relatively large pores. This was most dramatic in the spongy mesophyll (the primary pathway for gaseous CO_2_ diffusion within the leaf). Cell separation is an essential and ubiquitous component of leaf differentiation about which remarkably little is known, with most work in this area focusing on the dramatic events of abscission (Roberts *et al.*, 2002). Our data are consistent with the idea that, subsequent to cell division termination in leaf development, AGP19 plays a role in regulating the degree of cell separation that occurs to define the spongy mesophyll, and, moreover, that RBR1 plays an upstream role in regulating this process. Our understanding of exactly how AGPs function is speculative (Seifert and Roberts, 2007), but the work reported here identifies a potential link between a cell-cycle regulator (RBR1), a cell wall protein (AGP19) and a differentiation phenotype (cell separation in the mesophyll). Further characterization of the response of *AGP19* to altered *RBR1* expression is required to clarify the nature of this link, for example the extent to which AGP19 may rescue the RBRRNAi phenotype.

### Combined microCT imaging and chlorophyll fluorescence/gas exchange as a tool for analysis of the relationship of cell division and leaf form and function

Leaves have a specific histology that reflects the pattern of cell division and growth over time. The distribution of the resultant cell types generates identifiable tissues that perform specific functions so that the organ functions as a whole. This function may generally be described as the physiology of the organ, and, in the context of the leaf, encompasses activities such as photosynthesis and gas exchange. Various models relating leaf cellular architecture to photosynthesis have been generated, but a frequent limitation of these models has been the difficulty of relating 3D cellular architecture to the measured values of physiological processes, with various assumptions being made regarding gas flux (Parkhurst, 1994). We present here an integrated approach in which cell-cycle gene expression may be modulated in a target organ (the leaf), the 3D structural outcome at both the whole-organ and cellular level may be rapidly and non-invasively quantified by microCT, and the physiological parameters may be measured by combined fluorescence/gas exchange. We believe that this is an extremely powerful approach, with advances in microCT imaging especially allowing clearer, quantitative insights into the cellular outcome of altered cell-cycle gene expression. In the study reported here, this approach provided results indicating that, despite apparently quite major changes in cellular architecture, the plasticity of plant physiology/biochemistry is such that an essential function of the leaf (carbon assimilation) may be maintained at levels similar to normal. This is encouraging from an agronomic point of view, as it suggests that plants have an inherent capacity for increased efficiency in photosynthesis that may be selected or engineered (von Caemmerer and Evans, 2010). However, on the other hand, it suggests that our ability to exploit such potential may be limited by endogenous mechanisms that autoregulate leaf physiology towards a developmentally set level. Understanding how that level is set and to what extent the system may be re-modelled for agronomic advantage is a major challenge for the future.

## Experimental procedures

### Plant growth and gene induction

Arabidopsis seeds were kept at 4°C for 1 week before sowing. The *pOpOFF-RBR* seeds have been described previously (Kuwabara *et al.*, 2011). A pFAMA::RBRRNAi construct was generated by cloning a previously described RBRRNAi construct (Kuwabara *et al.*, 2011) into a pOpON vector (Wielopolska *et al.*, 2005) into which the pFAMA promoter sequence (Ohashi-Ito and Bergmann, 2006) had been cloned by Gateway-directed recombination. The pFAMA::RBRRNAi construct was introduced into Col–0 Arabidopsis plants by standard techniques (Clough and Bent, 1998), and T_3_ generation homozygous plants were obtained for analysis. Plants were grown in Conviron (http://www.conviron.com/) growth chambers in M3 soil under a 8 h light/16 h dark cycle at 22°C day/20°C night, 60% relative humidity. After germination, seedlings with four visible leaves were transplanted into individual pots. At 15 DAS, plants with six visible leaves were treated once on the apex with a drop (30 μl) of inducer, and allowed to grow for a further 25 days to maturity before analysis. The inducer solution contained 5 μM dexamethasone (DEX) in 0.005% v/v dimethylsulfoxide solution, with an equivalent volume of 0.005% v/v dimethylsulfoxide being used for controls.

### RNA and GUS reporter gene analysis

For quantitative RT–PCR, plants were collected at 15 days after sowing (*t *=* *0) and at intervals after induction/mock induction (24, 48 and 72 h). RNA was extracted using Trizol reagent (Invitrogen, http://www.lifetechnologies.com) according to the manufacturer's instructions. After DNAse treatment (TURBO DNA-free™, Ambion, http://www.lifetechnologies.com), RNA was reverse-transcribed using MLV reverse transcriptase (Promega, http://worldwide.promega.com). Quantitative PCR was performed in 96-well plates on an Applied Biosystems StepOnePlus PCR machine according to the manufacturer's instructions (http://www.lifetechnologies.com), with SYBR Green reagent (http://www.lifetechnologies.com). The PCR programme comprised denaturation for 10 min at 95°C, followed by 40 cycles of 95°C for 15 sec and 60°C for 1 min. All reactions were performed in triplicate with three independent biological replicates per sample. For analysis, normalized expression (ΔΔ*C*_T_) was calculated using Applied Biosystems StepOne software version 2.2. *C*_T_ values were normalized to the *C*_T_ values of an internal control, *AtPP2A* (*Protein Phosphatse2*). Primer sequences are listed in Table S1.

For GUS reporter gene expression, seedlings were immersed in ice-cold 90% v/v acetone for 10 min, then histochemistry was performed using the indigogenic substrate 5 bromo-4-chloro-3-indolyl glucuronide (0.5 mg ml^−1^), in 100 mm NaH_2_PO_4_ (pH 7.0) in the presence of ferrous/ferricyanide (5 mm) at 37°C overnight. Samples were rinsed in 100% v/v ethanol overnight, and observed under a Leica LZ10 stereomicroscope (http://www.leica-microsystems.com). Images were captured using a SPOT camera system (http://www.spotimaging.com/).

### Anthocyanin and chlorophyll content

For anthocyanin analysis, samples were snap-frozen in liquid N_2_, then ground in methanol/1% v/v HCl. Samples were kept in the dark at 4°C overnight. A one-fifth volume of Milli–Q H_2_O (Millipore, http://www.millipore.com/) was added, followed by a half volume of chloroform. After centrifugation (10 000 ***g***, 5 min), the supernatant was collected and the total volume made up to 800 μl by adding 60% methanol/1% v/v HCl and 40% Milli–Q H_2_O. Absorbance was read at 530 and 657 nm. For chlorophyll analysis, samples were extracted in 96% ethanol, and heated to 70°C in the dark until the tissue was white. Absorbance was read at 665 and 649 nm.

### Analysis of leaf growth, epidermal patterning and histology

For analysis of area, leaf 8 was removed and scanned, with cuts being made in the leaf perimeter as required to ensure a flat structure. For growth data, leaf 8 was dissected at various stages of development, fixed in acetic acid/ethanol (1:7 v/v), and images were taken using a Leica LX 12.5 stereomicroscope and SPOT camera. To image the epidermis, after fixation, the tissue was cleared (Kuwabara *et al.*, 2011) and then viewed using a BX51 microscope and camera (Olympus, http://www.olympus.co.uk). Analysis was performed using ImageJ (http://rsbweb.nih.gov/ij/), with images being captured from at least eight individual leaves for each treatment. For histology, leaves were fixed in ethanol/ acetic acid (80:20, v/v) overnight at 4°C, dehydrated in a progressive series of ethanol dilutions, and embedded in Technovit 7100 resin (Heraeus Kulzer GmbH, http://heraeus-kulzer.com). Sections (4 μm) were taken using a Leica RM 2145 microtome. After staining with toluidine blue (0.05% v/v), samples were observed under a BX51 microscope (Olympus).

### Micro X–ray computed tomography

Seven replicate WT and RBRRNAi leaves (both induced and control-treated) were used for X–ray microCT scanning. Each leaf was wrapped around a glass rod and placed in a 1.5 ml micro-centrifuge tube and held in place with a conical-shaped polystyrene bung to minimize movement of the sample during the scan. For analysis of the pFAMA::RBRRNAi leaves, the protocol was adapted to also include analysis of leaf discs (diameter 20 mm), with one disc being analysed per leaf. Each sample was scanned over 30 min using a Phoenix Nanotom 180NF X–ray computed tomography system (GE Sensing and Inspection Technologies GmbH, http://www.ge-mcs.com) fitted with a molybdenum transmission target at a maximum electron acceleration energy of 50 kV, a current of 210 μA, and 900 projection images were acquired. The detector size during the scan was 2304 × 2304 pixels, resulting in a spatial resolution of 4.5 μm. At this resolution, an image of 9 mm length from the tip of the leaf was acquired. Projection images were reconstructed using Datos|rec reconstruction software (GE Sensing and Inspection Technologies GmbH) using a filtered back-projection algorithm. Leaf morphology and intracellular air space were quantified using the automatic material calibration tool within Studio Max version 2.0 (Volume Graphics, http://www.volumegraphics.com), in which the background (air) and material (leaf) are differentiated based on the grey value of individual voxels, which relates to the X–ray attenuation and hence material density of the sample. The intracellular pore space was quantified within a region of interest (created from a mask fitted to the surface of the leaf) by summing the voxels defined as air from the calibration.

### Chlorophyll fluorescence and gas exchange analysis

Plants were placed in darkness for 30 min, and chlorophyll fluorescence was measured over a wide range of light intensities (200–1200 μmol m^−2^ sec^−1^) using a lab-based imaging system (Fluorimager, Technologica, http://www.technologica.co.uk/). To calculate light absorption, the flux of light reflected on and transmitted through leaves was measured under a white incident light source (Schott, http://www.schott.com). The light intensity was measured using a light meter and a quantum sensor (Li–189 and Q20806, LI-COR). For gas exchange, measurements of steady-state maximal rates of gas exchange were obtained using a portable open gas exchange system (LI–6400; LI–COR), equipped with a CO_2_ mixer, 30 × 20 mm chamber and red/blue LED light source (LI–6400-02B, LI-COR). The concentration of CO_2_ entering the cuvette was 400 μmol mol^−1^ air at a air flow rate of 200 μmol sec^−1^. The leaf vapour pressure deficit was maintained at 1.3 kPa ± 10%. The photosynthetically active photon flux density was set at 200, 400 and 1250 μmol m^−2^ sec^−1^, with 1250 μmol m^−2^ sec^−1^ having been determined to be saturating for photosynthesis based on preliminary light response curves. Prior to measurements, plants were pre-adapted to high light and a low vapour pressure deficit environment for a minimum of 15 min, and were then placed into a propagator lined with wet paper towels and lit by a Schott lamp, which generated a photosynthetically active photon flux density ≥1000 μmol m^−2^ sec^−1^ at rosette level. To correct gas exchange measurements for leaf area, a permanent marker was used to mark onto the leaf the location of the inner edges of the cuvette gaskets after each measurement was made. An image of the rosette and a steel ruler scale was then captured using a digital camera. ImageJ was used to measure the projected leaf area inside the cuvette. Each round of measurements included each of the four treatment types (WT or RBRRNAi, induced or mock-induced), with the order being shuffled so that two samples of the same type were never measured consecutively.

## Supporting Information

Movie S1 MicroCT imaging of a wild-type Arabidopsis leaf.

Figure S1 Growth curves of Arabidopsis leaves.

Figure S2 Transient GUS reporter gene expression following DEX induction of RBRRNAi seedlings.

Figure S3 Development of leaf porosity over time.

Figure S4 Total chlorophyll and anthocyanin content in induced WT and induced RBRRNAi leaves.

Table S1 List of quantitative RT–PCR primers.
