# Supplementary material for: Increased leaf mesophyll porosity following transient retinoblastoma-related protein silencing is revealed by microcomputed tomography imaging and leads to a system-level physiological response to the altered cell division pattern
Source: Plant J. 2013 Nov 11;76(6):914–29. doi: 10.1111/tpj.12342 (PMC4282533; doi:10.1111/tpj.12342)
Supplement: Figure S1 — Growth curves of Arabidopsis leaves. [file tpj0076-0914-SD2.pdf]

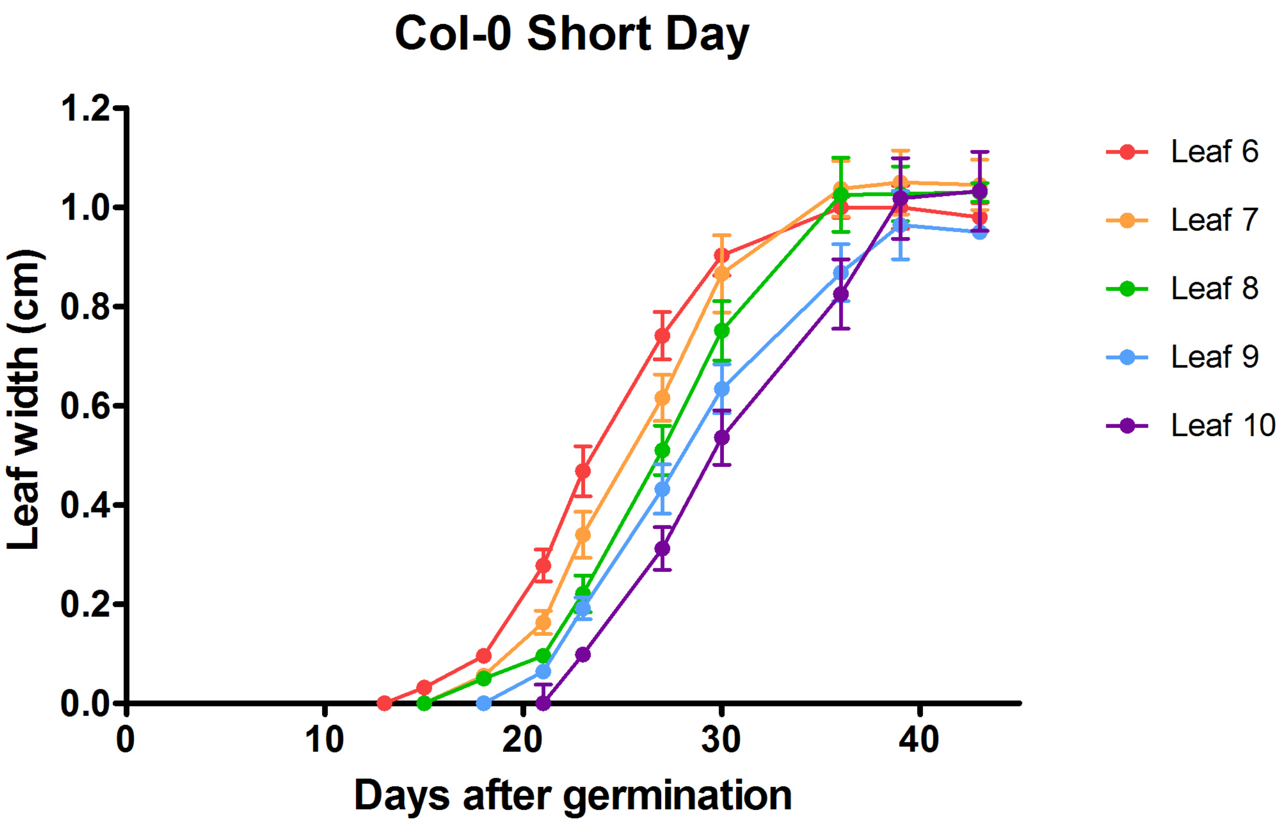

Supplementary Fig.1 Growth curves of Arabidopsis leaves.
Leaf width against time for leaf 6 to 10 (n = 8), error bars represent s.d.
Leaf 8 shows a consistent growth pattern with growth terminating before d40.
