# Supplementary material for: Increased leaf mesophyll porosity following transient retinoblastoma-related protein silencing is revealed by microcomputed tomography imaging and leads to a system-level physiological response to the altered cell division pattern
Source: Plant J. 2013 Nov 11;76(6):914–29. doi: 10.1111/tpj.12342 (PMC4282533; doi:10.1111/tpj.12342)
Supplement: Figure S2 — Transient GUS reporter gene expression following DEX induction of RBRRNAi seedlings. [file tpj0076-0914-SD3.pdf]

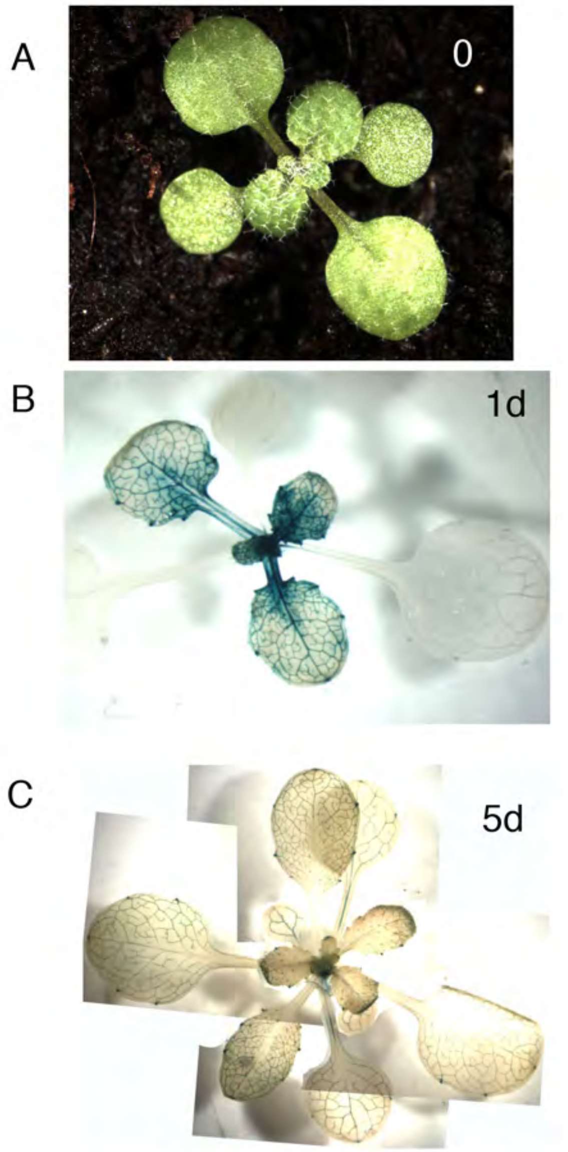


**Supplementary Fig. 2 Transient GUS reporter gene expression following dex induction of RBRRNAi seedlings**.

RBRRNAi seedlings at 15DAS (**A**) were induced with dex on the shoot apex. Within 24h reporter gene expression (blue) was visible in the leaves around the apex (**B**). By 5d after induction reporter gene expression was no longer visible (**C**).
