# Supplementary material for: Increased leaf mesophyll porosity following transient retinoblastoma-related protein silencing is revealed by microcomputed tomography imaging and leads to a system-level physiological response to the altered cell division pattern
Source: Plant J. 2013 Nov 11;76(6):914–29. doi: 10.1111/tpj.12342 (PMC4282533; doi:10.1111/tpj.12342)
Supplement: Figure S3 — Development of leaf porosity over time. [file tpj0076-0914-SD4.pdf]

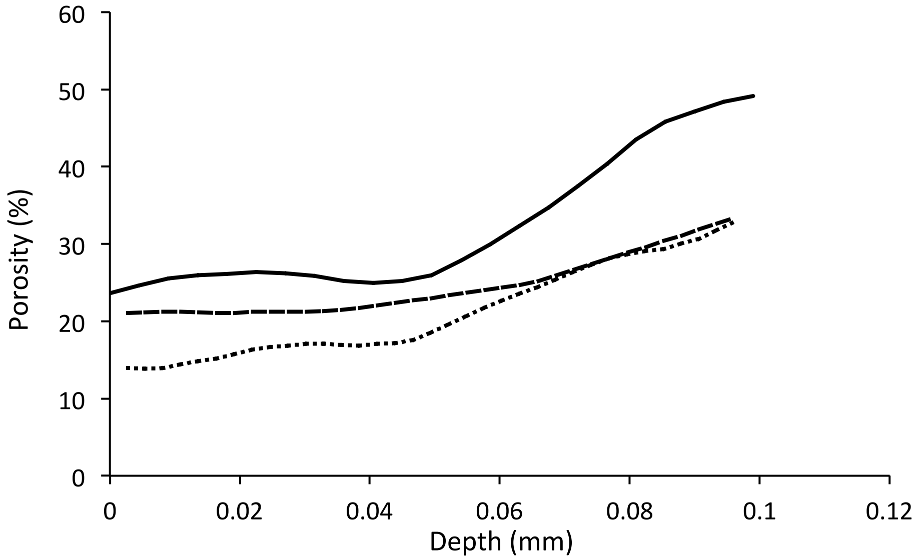

Supplementary Fig. S3. Development of leaf porosity with time. The graph shows mean leaf porosity across 0.1mm of the vertical axis (adaxial to abaxial) for WT leaves at d28 (dots), d35 (broken line) and d40 (intact line) (n=6). For clarity, variance is not shown. In all cases there is a gradient of porosity (with the abaxial side showing highest values) but porosity values are lowest at d28, with earlier time points (d21) indicating porosity values not distinguishable from system noise.
