# Supplementary material for: Increased leaf mesophyll porosity following transient retinoblastoma-related protein silencing is revealed by microcomputed tomography imaging and leads to a system-level physiological response to the altered cell division pattern
Source: Plant J. 2013 Nov 11;76(6):914–29. doi: 10.1111/tpj.12342 (PMC4282533; doi:10.1111/tpj.12342)
Supplement: Figure S4 — Total chlorophyll and anthocyanin content in induced WT and induced RBRRNAi leaves. [file tpj0076-0914-SD5.pdf]

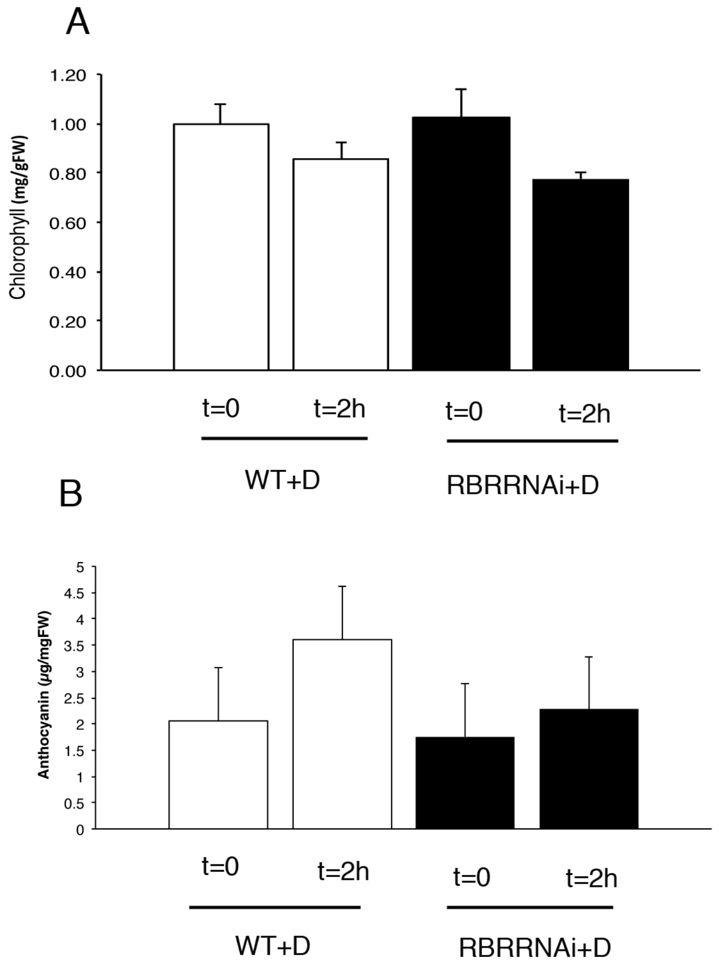

Supplementary Fig. S4. Total chlorophyll and anthocyanin content in induced WT and induced RBRRNAi leaves
