# Supplementary material for: Increased leaf mesophyll porosity following transient retinoblastoma-related protein silencing is revealed by microcomputed tomography imaging and leads to a system-level physiological response to the altered cell division pattern
Source: Plant J. 2013 Nov 11;76(6):914–29. doi: 10.1111/tpj.12342 (PMC4282533; doi:10.1111/tpj.12342)
Supplement: Table S1 — List of quantitative RT–PCR primers. [file tpj0076-0914-SD6.pdf]

Supplementary Table S1

List q-RT-PCR PRIMERS

Accesion number	Gene Name	Sequence 3' – 5'
At3g12280	RBR	TCATAAGTCGCCTGCTGCTAAG<br>CTGGTGTTGCTGCCAACTTG
At1g13320	PP2A	CAAGTGAACCAGGTTATTGGGA<br>ATAGCCAGACGTACTCTCCAG
At1g68725	AGP19	CCTACCAAGCACAAGAGAAAGCA<br>TCTGTTAGAACTGGAGGAGATGGA
At4g37450	AGP18	CCGCTACATTCCTCAACAC<br>CACCACCGACTGAATCTC
At2g23130	AGP17	CTCCTCATAAGCCTAAACCCACAT<br>ACAGGTGCTTCTACCGGAGTCT
At2g23430	KRP1	CGGAGCCGGAGAATTGTTTA<br>TCACCGACGACGGAGACA
At1g80080	TMM	GGCAATAATCTGACCGGAGA<br>TCACTGTCCCGGTTAACACA
At5g53210	SPCH	GCTGCTCTTGAAGATTTGGCT<br>CACTCAATTCCAATCTTGATGGTG
