# Supplementary material for: Increased leaf mesophyll porosity following transient retinoblastoma-related protein silencing is revealed by microcomputed tomography imaging and leads to a system-level physiological response to the altered cell division pattern
Source: Plant J. 2013 Nov 11;76(6):914–29. doi: 10.1111/tpj.12342 (PMC4282533; doi:10.1111/tpj.12342)
Supplement: Table S1 — List of quantitative RT–PCR primers. [file tpj0076-0914-SD8.docx]

**Supporting Information Legends**

**Supplementary Figure 1** Growth curves of Arabidopsis leaves. Leaf width against time for leaf 6 to 10 (n =8), error bars represent s.d. Leaf 8 shows a consistent growth pattern with growth terminating before 40 DAS.

**Supplementary Fig. 2** Transient GUS reporter gene expression following dex induction of RBRRNAi seedlings. RBRRNAi seedlings at 15 DAS (**A**) were induced with dex on the shoot apex. Within 24 h reporter gene expression (blue) was visible in the leaves around the apex (**B**). By 5 d after induction reporter gene expression was no longer visible (**C**).

**Supplementary Fig. S3**. Development of leaf porosity with time. The graph shows mean leaf porosity across 0.1mm of the vertical axis (adaxial to abaxial) for WT leaves at d28 (dots), d35 (dashed line) and d40 (solid line) (n=6). For clarity, variance is not shown. In all cases there is a gradient of porosity (with the abaxial side showing highest values) but porosity values are lowest at d28, with earlier time points (d21) indicating porosity values not distinguishable from system noise.

**Supplementary Fig. S4**. Total chlorophyll and anthocyanin content in induced WT and induced RBRRNAi leaves.

**Supplementary Table S1** List q-RT-PCR primers.

**Supplementary movie** MicroCt imaging of wild-type Arabidopsis leaf.
